# The Expression of c-Myb Correlates with the Levels of Rhabdomyosarcoma-specific Marker Myogenin

**DOI:** 10.1038/srep15090

**Published:** 2015-10-14

**Authors:** Petr Kaspar, Martina Zikova, Petr Bartunek, Jaroslav Sterba, Hynek Strnad, Leos Kren, Radislav Sedlacek

**Affiliations:** 1 Laboratory of Transgenic Models of Diseases, Institute of Molecular Genetics of the ASCR, v.v.i., Prague, Czech Republic; 2 Laboratory of Cell Differentiation, Institute of Molecular Genetics of the ASCR, v.v.i., Prague, Czech Republic; 3 The University Hospital Brno, Brno, Czech Republic; 4 Laboratory of Genomics and Bioinformatics, Institute of Molecular Genetics of the ASCR, v.v.i., Prague, Czech Republic

## Abstract

The transcription factor c-Myb is required for modulation of progenitor cells in several tissues, including skeletal muscle and its upregulation is observed in many human malignancies. Rhabdomyosarcomas (RMS) are a heterogeneous group of mesodermal tumors with features of developing skeletal muscle. Several miRNAs are downregulated in RMS, including miR-150, a negative regulator of c-Myb expression. Using the C2C12 myoblast cell line, a cellular model of skeletal muscle differentiation, we showed that miR-150 controls c-Myb expression mainly at the level of translation. We hypothesized that a similar mechanism of c-Myb regulation operates in RMS tumors. We examined expression of c-Myb by immunohistochemistry and revealed c-Myb positivity in alveolar and embryonal tumors, the two most common subgroups of RMS. Furthermore, we showed direct correlation between c-Myb production and myogenin expression. Interestingly, high myogenin levels indicate poor prognosis in RMS patients. c-Myb could, therefore, contribute to the tumor phenotype by executing its inhibitory role in skeletal muscle differentiation. We also showed that c-Myb protein is abundant in migratory C2C12 myoblasts and its ectopic expression potentiates cell motility. In summary, our results implicate that metastatic properties of some RMS subtypes might be linked to c-Myb function.

The transcription factor c-Myb is required for the regulation of progenitor cells in several tissues, including the hematopoietic system^1^,^2^, the adult brain^3^, and colonic crypts^4^. It plays a role in progenitor production, maintaining their proliferation, migration, or lineage commitment. c-Myb expression generally declines as progenitor cells differentiate. In fact, constitutive overexpression of c-Myb in immature myeloid and erythroid cell lines blocks their differentiation^5^,^6^. c-Myb is also implicated in differentiation of smooth muscle cells^7^ and may play a role in skin development and wound healing^8^. Using C2C12 cells and myoblasts derived from ex-vivo cultured myofibers it was shown that c-Myb is expressed in skeletal muscle progenitor cells and turned off in terminally differentiated cells. Moreover, it was demonstrated that skeletal muscle differentiation is blocked by constitutively expressed c-Myb^9^. c-Myb activity is tightly regulated at different levels, including downregulation by miRNAs. miR-150^10^ and miR-126^11^ were shown to inhibit c-Myb expression *in vivo*. c-Myb is overexpressed in many human malignancies^12^,^13^. Several studies also showed that aberrant miRNA expression is associated with a variety of cancers. Low levels of miR-150 were detected in some types of cancer that exhibit high-levels of c-Myb expression, such as colorectal cancer^14^, chronic myeloid leukemia^15^, and acute lymphoblastic leukemia^16^. It is suggested that the decrease in abundance or absence of negative regulator miR-150 could result in activation of c-Myb in these tumors.

RMS is the most common soft tissue sarcoma in children accounting for 5% to 10% of all pediatric malignancies^17^. RMS can be classified into several histologic subgroups: embryonal rhabdomyosarcoma (ERMS), which has embryonal, botryoid, and spindle cell subtypes; alveolar rhabdomyosarcoma (ARMS); pleomorphic rhabdomyosarcoma; and spindle cell/sclerosing rhabdomyosarcoma^18^. RMS are a group of mesodermal cancers that express multiple genes characteristic of muscle cell differentiation, such as myogenic transcription factors MyoD and myogenin^19^ as well as other skeletal muscle-specific markers such as desmin, actins, myosins, and creatine kinases^20^,^21^. Expression of skeletal muscle-specific markers suggests commitment to the skeletal muscle lineage; however, RMS instead exhibit defective skeletal muscle differentiation^22^. Myogenin is often used as a specific marker of RMS^23^. Diffuse expression in tumor cells (more than 80% cells with positive nuclear staining for myogenin) is a poor prognostic factor independent of RMS histologic subtype^24^. Using a zebrafish model of ERMS it was shown that myogenin-positive ERMS cells are highly migratory, able to enter vasculature and infiltrate blood vessels, and are also the first to populate new areas of tumor growth^25^.

Several miRNas are downregulated in ARMS and ERMS compared to normal skeletal muscle, including miR-150^26^. As c-Myb expression could be elevated in RMS in consequence of low miR-150 levels, and contribute to abortive skeletal differentiation, we examined RMS for c-Myb expression.

We found that c-Myb is expressed in myogenin-positive ARMS and ERMS tumor specimens. Moreover, we show that c-Myb protein and myogenin colocalize in tumor cell nuclei, however, c-Myb expression is more abundant and is also detected in some myogenin-negative cells. Its expression can therefore inhibit skeletal muscle differentiation in myogenin-positive tumor cells and support tumorigenesis. Our results also show that c-Myb plays a role in migration of C2C12 cells and could be implicated in migratory ERMS cells that were shown to express myogenin.

## Results

### Microarray data reveals correlation between c-myb and myogenin expression

**Figure 1:**
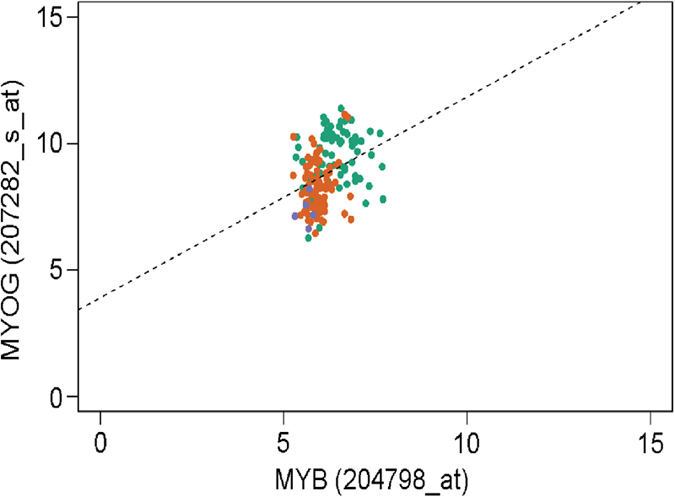
Spearman’s rank correlation for the determination of co-expression of c-myb and myogenin. For c-myb we analyzed data from probe set MYB, 204798_at and for myogenin from probe set MYOG, 207282_s_at^27^. Blue dots represent undifferentiated sarcoma, orange dots represent ERMS and green dots ARMS.

We performed analysis of a publicly available DNA microarray data on 120 RMS tumor specimens reported by Davicioni *et al.*^27^ and analyzed the data for c-myb expression, expression of myogenic regulatory factors: MyoD, myogenin, Myf5 and MRF4 (Myf 6), selected muscle-specific genes ACTN2, MYH8, TNNC2, CKM inhibited by c-Myb during myogenic differentiation of the C2C12 myoblast cell line^9^, and several other genes that are regulated by c-Myb, specifically: Slug^28^, cathepsin D, matrix metalloproteinases 1/9^29^, Bcl-2, c-Kit, cyclinB1, CD34, mim1^13^, CXCR4^30^, and Myb-binding protein 1A. The GeneChip® Human Genome U133A 2.0 Array used by Davicioni *et al.* provides two different probe sets to evaluate c-myb expression. We found variable c-myb expression among tumor tissues from low to moderate levels with probe set 204798_at, matching the 3′ UTR of c-myb (Supplementary Fig. 1) (the second, negative probe set 2015152_at, matched intron number 8). Next, we used Spearman’s rank correlation to compare the c-myb expression profile determined by probe set 204798_at with profiles of other analyzed genes to examine potential correlations in gene expression (Spearman’s rank correlation coefficient ρ, Supplementary Fig. 1). Moderate correlation was identified for myogenin with ρ = 0.404, p-value = 8.41e–08 (Fig. 1), indicating a similar expression pattern. Moreover, for MyoD, we found ρ = 0.311, p-value = 4.31e–05, indicating weak correlation (as for the correlation between MyoD1 and myogenin: ρ = 0.437, p-value = 3.55e–09). As RMS cases are diagnosed by expression of myogenin and MyoD, we speculated that c-myb could be implicated in RMS tumorigenesis.

### miR-150 preferentially targets c-Myb protein levels in C2C12 myoblasts

**Figure 2:**
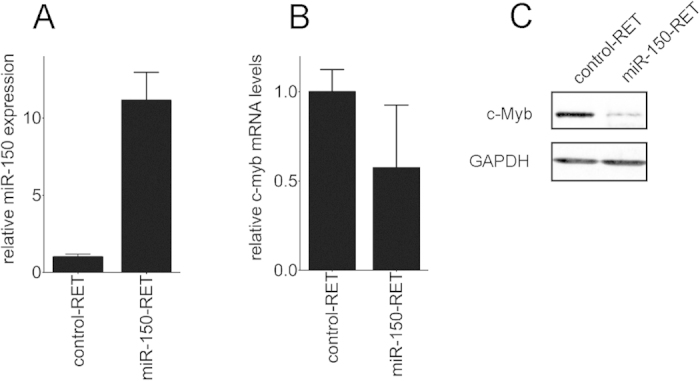
miR-150 acts as an efficient inhibitor of translation of c-myb mRNA. Subconfluent control C2C12 myoblasts expressing empty retrovirus (control-RET) or miR-150 expressing cells (miR-150-RET) were analyzed for: miR-150 expression (**A**) and c-myb mRNA levels (**B**) in miR-150-RET-expressing cells relative to control RET-expressing cells by qPCR, c-Myb protein levels were determined by Western blotting (**C**). Data were analyzed using GraphPad Prism software with t-test data analysis procedure.

Another indicator of c-Myb possible involvement in tumorigenesis may be low-levels of its negative regulator miR-150, detected in RMS. As c-myb mRNA levels are not significantly increased in RMS (DNA microarray data), we hypothesized that miR-150 could predominantly interfere with c-myb translation. Since RMS have features of developing skeletal muscle, we investigated the mechanism of action of miR-150 on c-myb expression using the C2C12 myoblast cell line, a model of skeletal muscle development. Since it was documented that C2C12 cells express low levels of miR-150^31^, we increased the miR-150 levels in the cells and analyzed their effect on c-myb. We infected C2C12 cells with miR-150-expressing retrovirus (miR-150-RET) and control empty retrovirus (control-RET). After exposure to the retroviruses for 24 hours, the cells were sorted for eGFP; positive cells were collected and cultured in growth medium. We found that in miR-150-RET-infected cells, expression of miR-150 was increased more than 10 times compared to cells infected with the empty retrovirus (Fig. 2A), c-myb mRNA levels were decreased to 50% of control levels (Fig. 2B), while c-Myb protein levels were almost extinguished (Fig. 2C). These results indicate that miR-150 preferentially targets c-Myb translation. Low levels of miR-150 in RMS could hence lead to more efficient mRNA translation and accumulation of c-Myb protein.

### c-Myb and myogenin protein levels correlate in RMS

Next, we examined a RMS tissue microarray (TMA) for c-Myb expression by immunohistochemistry (IHC) analysing Leiomyosarcoma and rhabdomyosarcoma TMA (#S0751, US Biomax, Inc.). The assay contained 18 cases of leiomyosarcoma and 18 cases of rhabdomyosarcoma, duplicate cores per case, and cardiac and smooth muscle as control samples. We identified c-Myb expression in some tumor specimens. Positive staining was detected in both cores in the case of ARMS from striated muscle, in the case of ERMS from the tongue and testis, and low expression was detected in ERMS from retroperitoneum. Negatively stained were cases of ARMS from soft tissue, ERMS from pelvic cavity and abdominal cavity, one case of RMS from striated muscle, both cases of spindle RMS, all eight cases of pleomorphic RMS and all 18 cases of leimyosarcoma (Supplementary Fig. 2). The results of immunostaining indicate expression of c-Myb in some cases of ARMS and ERMS but not in pleomorphic RMS.

**Figure 3:**
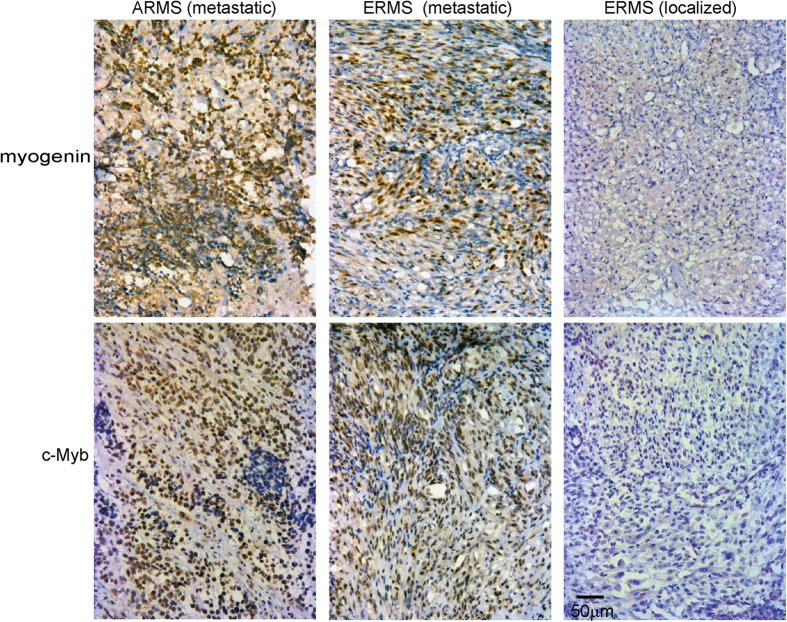
Immunohistochemical staining for c-Myb and myogenin in metastatic ARMS and ERMS and localized ERMS. We show analysis of ARMS tumor sample 15 with metastatic phenotype, ERMS tumor sample 10 with metastatic phenotype and ERMS tumor sample 19 with localized phenotype.

**Figure 4:**
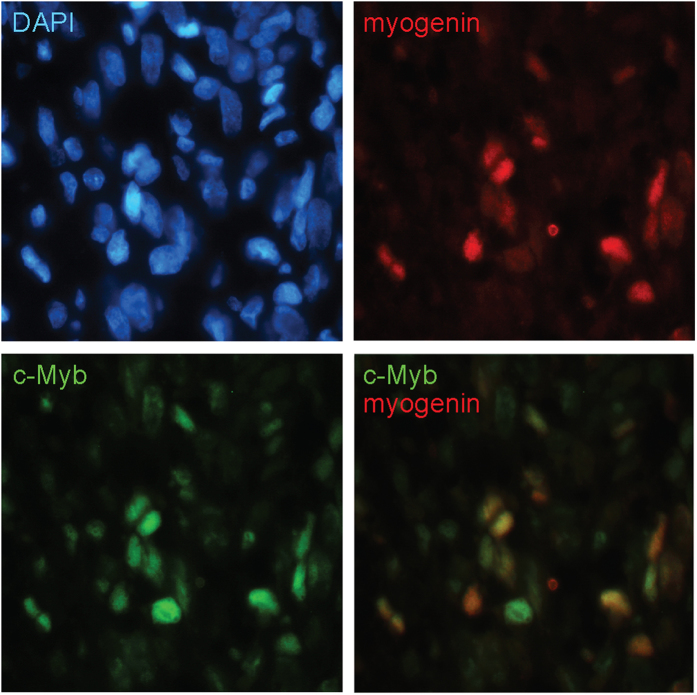
Immunofluorescence staining for c-Myb and myogenin in an ARMS case. We analyzed tumor sample 15, with metastatic phenotype. Nuclei were counterstained by DAPI.

To gain insight into the correlation of c-Myb and myogenin expression in RMS, a total of 19 tumors were evaluated. RMS tissue slides were provided by the pathologists from The University Hospital Brno. These included seven cases of ARMS, one case of mixed ARMS/ERMS, and eleven cases of ERMS. Formalin-fixed paraffin-embedded blocks were immonostained for c-Myb and myogenin simultaneously under identical conditions (Fig. 3). Immunopositive cells taken from five representative pictures were manually tagged and counted using an image analysis system (NIH Image J software). The results of scoring of staining were summarized in Supplementary Table 1, some clinical and histopathologic information of analyzed RMS tumor samples were also presented. We noticed that c-Myb is slightly more abundant than myogenin in most tumor samples. The results of scoring of staining presented in Supplementary Table 1 were plotted. We plotted separately data for localized tumors and metastatic tumors (Supplementary Fig. 4A,B). We showed that percentage of both c-Myb and myogenin positive cells was reduced in localized tumors compared to metastatic tumors. When results of scoring from Supplementary Table 1 were analyzed by Spearman’s rank correlation (Supplementary Fig. 4C) very strong correlation was identified (ρ = 0.94, p-value = 0.0001225) indicating that c-Myb and myogenin expression correlates in all examined tumor samples. We then investigated whether these genes were co-expressed within the cell. To this end we performed immunofluorescence analysis of the same set of RMS tissue slides. We found that c-Myb colocalized with myogenin in all tumor specimens tested (Fig. 4). Moreover, c-Myb expression was not restricted to myogenin-positive cells, but was also detected in some cells stained negative for myogenin. Altogether, we found that c-Myb was expressed in RMS tumor cells marked by myogenin expression.

### c-Myb is expressed in RMS cell lines

We have shown that c-Myb is expressed in RMS tumors. Next, we were interested whether c-Myb is also expressed in RMS cell lines. We have chosen two representative cell lines: ERMS was represented by RD and ARMS was represented by RH30. We evaluated c-Myb expression by western blotting of growing cells and differentiating for two days and four days (Supplementary Fig. 3). c-Myb expression was detected in both RMS cell lines in all time points.

### c-Myb supports migration of C2C12 myoblast cells

**Figure 5:**
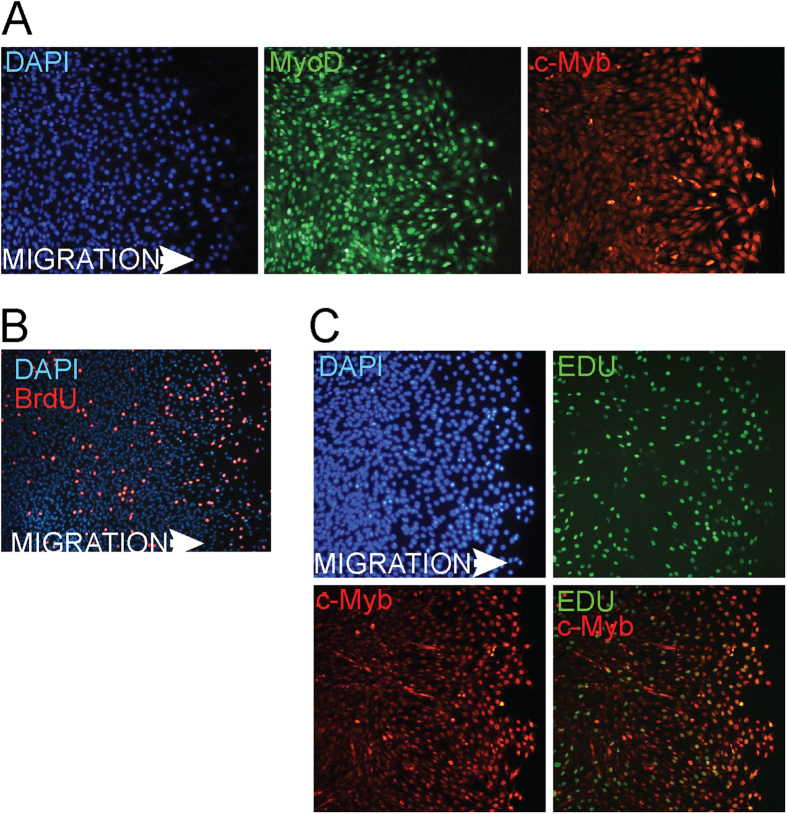
Migrating C2C12 myoblasts express c-Myb. Wound healing migration assays of confluent C2C12 myoblasts in growth conditions were performed. Some cells were scraped off using a pipette tip to obtain a cell-free area. The wounded cell cultures were incubated for 24 hours to allow cells to migrate into the acellular area and analyzed for expression of MyoD, a C2C12 myoblasts marker, (**A**) and c-Myb (**A,C**) by immunuflorescence. Cell proliferation was assessed by BrdU (**B**) or EdU (**C**) staining. Nuclei were counterstained by DAPI. The representative results of three independent experiments are shown.

**Figure 6:**
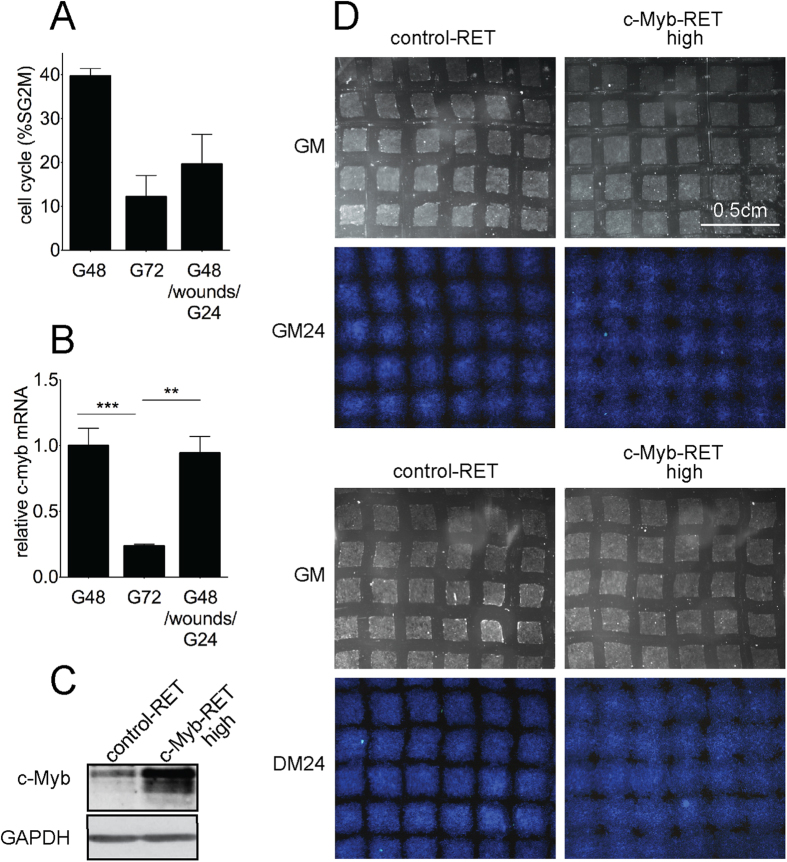
The predominant cause of elevated c-Myb expression is the cell migration process itself. After cultivation for 48 hours in growth medium (G48), confluent cells were subjected to the wound healing assay. Wounds were created by a comb. Wounded cell cultures were incubated for an additional 24 hours (G48/wounds/G24) and analyzed, control cell cultures were not wounded and cultivated for a total of 72 hours (G72) before analysis. Cell cycle progression was determined by flow cytometry (**A**) and c-myb mRNA levels relative to c-myb levels in G48 cells were determined by qPCR (**P  < 0.01, ***P < 0.001). Data were analyzed using GraphPad Prism software with t-test data analysis procedure (**B**). Migration of control and c-Myb-overexpressing C2C12 cells. Cells were infected with empty retrovirus (control-RET) or retrovirus expressing c-Myb and eGFP (c-Myb-RET) and sorted for high eGFP to obtain cell cultures expressing high levels of c-Myb (c-Myb_high_-RET). Sorted, confluent cell cultures were analyzed by Western blotting (**C**). Wound healing assay of control C2C12 cells and c-Myb overexpressing cells. Confluent cell cultures in growth medium (GM) were wounded and allowed to migrate for 24 hours in either conditioned growth medium (GM24) or differentiating medium (DM24), fixed, nuclei were stained with DAPI (**D**). Representative results of three independent experiments are shown.

Recently it was shown that myogenin also denotes migratory ERMS cells. We accordingly examined whether c-Myb could play a role in cell migration using C2C12 myoblast cells. To examine whether c-Myb is involved in cell migration, we performed wound healing experiments in C2C12 cells. Immunofluorescence staining proved elevated c-Myb expression in the majority of cells migrating into the wound, indicating that c-Myb is expressed in migratory C2C12 cells (Fig. 5A,C). However, migratory cells simultaneously re-entered the cell cycle as indicated by BrdU staining (Fig. 5B). The elevated c-Myb levels could thus be in response to cell cycle activation as we demonstrated before that c-Myb expression coincided with the proliferation rate of C2C12 cells^32^. Indeed, some c-Myb-positive migratory cells entered S phase as indicated by colocalization with EdU (Fig. 5C), and the remaining c-Myb-positive cells could be entering G_1_/S and G_2_/M phase transition, since it was shown that c-Myb may play a role in both these processes^33^,^34^. To discriminate between the contribution of cell cycle progression to c-Myb induction and the potential influence of the migration process itself, we performed the wound healing experiment with a different experimental set up. Confluent C2C12 cells were wounded by a comb, creating a series of nine mutually perpendicular cuts forming a total of 81 square areas (~150-μm^2^) containing confluent cells separated by 100-μm wide acellular areas (35-mm culture dish). Cells were allowed to migrate into the wounds and after 24 hours were harvested and analyzed. We performed cell cycle analysis by flow cytometry (Fig. 6A) and determined c-myb expression levels by qPCR (Fig. 6B). Analysis of control, unwounded C2C12 cells proved that c-myb expression levels coincided with cell proliferation. As the cell density increased, cells continued to slow down both the proliferation rate (from 40% to 12% cells in SG_2_/M phase after 72 hours of cultivation) and c-myb mRNA levels (to a quarter of its original levels). Interestingly, wounded C2C12 cells migrating into the wounds for 24 hours re-entered the cell cycle (20% cells in SG_2_/M phase) and c-myb expression reached almost the same levels as before creating wounds when the cell proliferation rate was high (40% SG_2_/M).

Our results indicate that the elevated expression of c-myb observed during migration of C2C12 cells is not in consequence of partial cell-cycle re-entry accompanying migration, but migration process itself is the principal factor.

To confirm this, we overexpressed c-Myb in C2C12 cells and estimated the migration capacity of cells. We infected C2C12 cells with c-Myb-expressing retrovirus (c-Myb-RET) or empty retrovirus. After exposure to retroviruses, confluent cells expressing high levels of c-Myb (confirmed by Western blotting, Fig. 6C) were subjected to the wound healing experiment in either growth or differentiation conditions. After 24 hours, C2C12 cells expressing high levels of c-Myb completely filled wounded area, irrespective of culture conditions, while the wounds in the culture of mock-infected cells were still apparent (Fig. 6D). Moreover, cells did not proliferate in the differentiation medium irrespective of c-Myb expression, but were able to migrate when c-Myb was expressed. Only 6% of cells infected with empty retrovirus entered SG_2_/M phase after 24 hours of cultures in the differentiation medium and 7% of cells when c-Myb was overexpressed. If cells migrated into the wounds in differentiation medium instead, the number of cells in SG_2_/M was almost equal: 7% of cells in SG_2_/M for mock-infected cells and 8% in SG_2_/M for cells overexpressing c-Myb.

As for the mechanism of c-Myb activation of cell migration, it was shown that Slug, which is involved in epithelial to mesenchymal transition (EMT), is regulated by c-Myb^28^. We therefore speculated that EMT could also be involved in migratory C2C12 cells. However, qPCR analysis did not confirm activation of Slug in the migrating cells.

To summarize, the wound healing experiments proved that c-Myb is involved in the migration of C2C12 myoblasts. Furthermore, we showed that elevated levels of c-Myb significantly increased the migratory capacity of cells.

## Discussion

Our previous results showed that c-Myb expression is regulated during the skeletal muscle differentiation. c-Myb is expressed in activated satellite cells. Its expression continues in proliferating myoblasts and is finally downregulated in multinucleated myotubes. Furthermore, we showed that continuous expression of c-Myb prevents formation of myotubes^9^. RMS cells show signs of skeletal muscle development, but are not capable to terminally differentiate and to form myotubes. Therefore, we asked whether c-Myb is expressed in RMS because it could contribute to the cells incapacity to differentiate. Moreover, miR-150, an inhibitor of c-Myb, is downregulated in ARMS and ERMS. We re-analyzed DNA microarray data reported by Davicioni *et al.*^27^ and identified variable expression of c-myb mRNA among tumors, spanning from low to moderate levels implying also low to moderate levels of c-Myb protein. However, some experiments indicate that the linear relationship between c-myb mRNA and protein levels is contentious. For instance, hematopoietic stem cells differing in c-Myb protein levels more than 2-fold exhibit no more than 1.3-fold difference in c-myb mRNA levels^35^. In the study reported by Lu *et al.*^36^ it was shown that despite similar c-myb mRNA levels in megakaryocyte-erythrocyte progenitors, miR-150 expression in the megakaryocytic lineage drives differentiation toward megakaryocytes by targeting c-myb translation. Therefore, we investigated the mechanism of action of miR-150 on c-myb expression in skeletal muscle using C2C12 cells. We found that protein translation was also the major target of miR-150 in these cells. Thus, we hypothesized that a similar mechanism of c-Myb regulation might operate in RMS tumors, which are marked by low miR-150 levels. Because of low levels of miR-150 more efficient translation of c-myb mRNA could be anticipated.

Interestingly, we found that some tumors with increased levels of c-myb mRNA also express higher levels of myogenin. The expression of myogenin was shown to be specific for rhabdomyoblastic differentiation, which makes it a useful marker in diagnosis^24^.

Next, we immunostained leiomyosarcoma and rhabdomyosarcoma tissue microarrays to investigate whether c-Myb protein was expressed. All leimyosarcoma samples were negative for c-Myb. Leimyosarcoma is a tumor arising from smooth muscle tissue and it was shown that c-Myb plays an important role in smooth muscle cell differentiation^7^. All cases of pleomorphic RMS were also negative for c-Myb. It is known that pleomorphic RMS occurs almost exclusively in adults^37^, in contrast to ARMS and ERMS, the two most common subgroups of RMSs found in children.

Commercially available tissue microarrays, however, provide just limited numbers of ARMS and ERMS. Of two ARMS cases spotted on array from US Biomax, the positivity for c-Myb was detected in one case only; of seven cases of ERMS examined, three cases were positive. To gain insight into the correlation of c-Myb and myogenin expression in ARMS and ERMS we evaluated an additional 19 tumors. We showed that percentage of both c-Myb and myogenin positive cells was reduced in localized tumors compared to metastatic tumors. Moreover, we observed strong correlation in the expression of c-Myb and myogenin in RMS and found that both genes were predominantly co-expressed. As myogenin marks RMS tumor cells, c-Myb co-expression could enhance RMS tumorigenesis by inhibiting differentiation of tumor cells.

Myogenin immunohistochemical positivity found in RMS cells distinguishes patients with poor clinical outcome, suggesting that myogenin-positive cells have a unique role in RMS progression and metastasis^24^. Using the transgenic zebrafish model of ERMS it was shown that myogenin-positive ERMS cells are highly migratory and precede recruitment of ERMS propagating cells in new colonized areas of tumor growth^25^.

In our study, we also showed that c-Myb is expressed in migratory C2C12 myoblast cells, and we proved that forced expression of c-Myb increases the migration capacity of cells even under differentiating conditions when migrating cells do not enter the cell cycle. However, the c-Myb target gene involved in EMT, Slug, was not induced in the migrating cells. We speculate that C2C12 cells, which are of mesenchymal origin, have already acquired the migratory mesenchymal phenotype and c-Myb, therefore, could induce migration by another mechanism.

To conclude, we proved that c-Myb is expressed in myogenin-positive RMS cells and could enhance their tumorigenesis by inhibiting differentiation and/or supporting their migration.

## Material and Methods

### qRT-PCR

qRT-PCR reactions were performed in triplicate using FastStart SYBR Green Master (Roche Applied Science, IN, USA) and analyzed by using the Light Cycler® 480 Instrument II (Roche Applied Science). GAPDH was used to normalize the RNA content of samples. The primers for c-myb were: 5′-AGATGAAGACAATGTCCTCAAAGCC-3′ and 5′-CATGACCAGAGTTCGAGCTGAGAA-3′; the primers for Slug were: 5′-TCTGTGGCAAGGCTTTCTCCAG-3′ and 5′-TGCAGATGTGCCCTCAGGTTTG-3′.To quantify miR-150, qRT-PCR was performed in triplicates using TaqMan MicroRNA assays (Applied Biosystems), assay ID: 000473, according to manufacturer’s instructions. Relative expression of miR-150 was normalized to corresponding U6 snRNA values, (assay ID: 001973).

### Western blotting

Western blotting was performed as we previously described^32^. Cell extracts were analyzed by monoclonal anti-Myb antibody (#05–175, clone1–1, Millipore) at dilution 1:500 or by monoclonal anti-GAPDH antibody (#GTX30666, GeneTex) at dilution 1:2000 according to the manufacturer’s instructions.

### Cell culture

The C2C12 mouse myoblast cell line was obtained from ATCC and cultured as recommended. Cells were maintained in growth medium and differentiated in low serum medium as described in detail elsewhere^32^. Fluorescent labelling of proliferating cell was done by BrdU staining or by using Click-iT® EdU Alexa Fluor® 488 Imaging Kit (Life Technologies). Immunofluorescence staining was performed as described^9^. Cell cycle progression was analyzed by flow cytometry. For DNA constructs, c-myb-expressing retrovirus pMSCV-cMyb-IRES-eGFP (c-Myb-RET) and miR-150-expressing retrovirus pMSCV-miR-150-IRES-eGFP (miR-150-RET), expressing the particular gene as a bicistronic transcript with eGFP, were described previously^9^. The infection of cells with retroviruses and cell sorting for eGFP were performed as described in^9^. To obtain cells with high c-Myb expression, C2C12 cells were infected with c-myb-expessing retrovirus (c-Myb-RET), sorted for high eGFP levels, positive cells were collected and cultured in growth conditions. RMS cell lines RD and RH30 were obtained from ATCC and cultured as recommended. To induce differentiation, 10% fetal bovine serum was replaced by 2%horse serum.

### Wound healing

Confluent C2C12 cells cultured in growth medium for 48 hours were subjected to the wound healing assay. To create acellular areas, a pipette tip or a comb was used to scrape off some cells to create a total of eighty one 150-μm^2^ square areas containing confluent cells separated by 100-μm wide acellular areas. The medium containing detached cells was then removed and replaced with conditioned medium from the same unwounded confluent cell culture. Alternatively, the differentiation medium (DM) was used. The exchange of medium was also done for control, unwounded cells to provide the same culture conditions. The wounded cell cultures were incubated for 24 hours to allow cells to migrate into the acellular area and analyzed.

### Immunohistochemistry

RMS tissue glass slides retrieved from archives were provided by the pathologists from The University Hospital Brno. Immunohistochemistry was carried out on 6-μm thick sections of tissue that were transferred to SuperFrost Plus slides. Slides were dewaxed and immersed in a steam bath (20 min, 10 mM citrate buffer, pH 6) for antigen retrieval. Afterwards the slides were washed in PBS and incubated with 5% goat nonimmune serum (Jackson ImmunoResearch) for 1 h at RT to block nonspecific binding. The primary antibodies used were rabbit polyclonal anti-MYB antibody (1:200, LSBio, LS-B5315) and mouse monoclonal anti-myogenin antibody (1:50, Santa Cruz, SC-12732). Staining was visualized using the avidin-biotin method using diaminobenzidine (DAB) as the chromogen. Slides were developed in DAB for exactly the same time to achieve the same intensity of background staining and counterstained with hematoxylin. Photographs were taken with a Leica DM2000 microscope and processed using Leica Application Suite (LAS) v4.3.0 and Adobe Photoshop software.

For immunofluorescence staining, secondary detection was performed using a goat anti-rabbit biotin conjugate followed by streptavidin-Alexa Fluor 488 and goat anti-mouse Alexa 568. Sections were mounted in ProLong Gold antifade reagent with DAPI (Life Technologies) to counterstain DNA.

## Additional Information

**How to cite this article**: Kaspar, P. *et al.* The Expression of c-Myb Correlates with the Levels of Rhabdomyosarcoma-specific Marker Myogenin. *Sci. Rep.* **5**, 15090; doi: 10.1038/srep15090 (2015).

## Supplementary Material

Supplementary Information
