# Supplementary material for: The Expression of c-Myb Correlates with the Levels of Rhabdomyosarcoma-specific Marker Myogenin
Source: Sci Rep. 2015 Oct 14;5:15090. doi: 10.1038/srep15090 (PMC4604482; doi:10.1038/srep15090)
Supplement: Supplementary Information [file srep15090-s1.pdf]

The Expression of c-Myb Correlates with the Levels of Rhabdomyosarcoma-specific Marker Myogenin

Petr Kaspar[1]*, Martina Zikova[2], Petr Bartunek[2], Jaroslav Sterba[3], Hynek Strnad[4], Leos Kren[3], Radislav Sedlacek[1]

[1] Laboratory of Transgenic Models of Diseases, Institute of Molecular Genetics AS CR, v.v.i., Prague, Czech Republic

[2] Laboratory of Cell Differentiation, Institute of Molecular Genetics AS CR, v.v.i., Prague, Czech Republic

[3] The University Hospital Brno, Brno, Czech Republic

[4] Laboratory of Genomics and Bioinformatics, Institute of Molecular Genetics AS CR, v.v.i., Prague, Czech Republic

*Corresponding author

E-mail: petr.kaspar@img.cas.cz

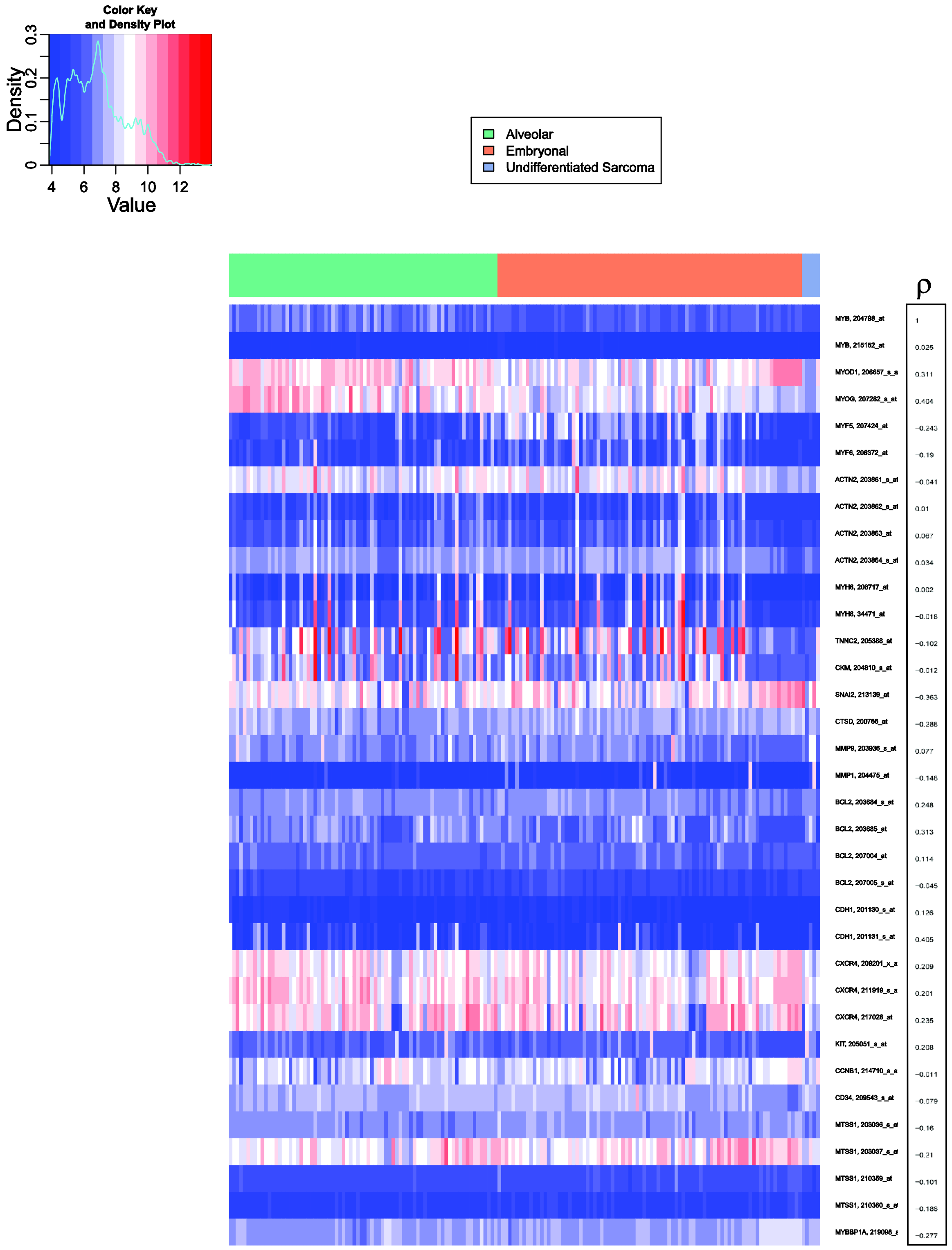

Supplementary Fig.1: Heatmap of gene expression of c-Myb-related genes and myogenic regulatory factors. Reanalysis of published DNA microarray data from 120 RMS tumor specimens reported by Davicioni et al.(28). Spearman's rank correlation coefficient ρ is indicated for correlation between c-Myb (probe set MYB, 204798_at) and the particular gene.

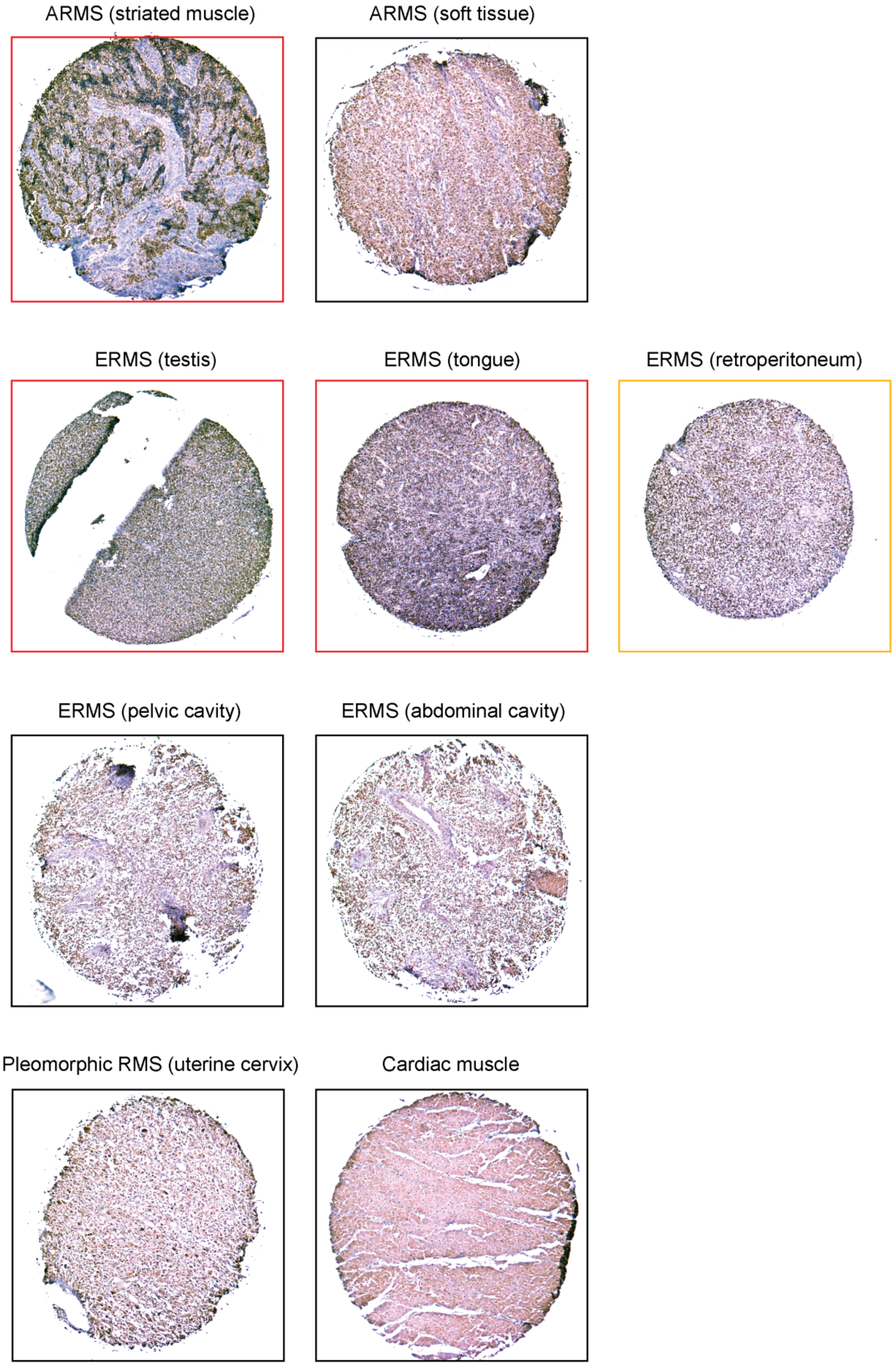

Supplementary Fig.2: Immunohistochemical staining of RMS tissue microarray (#S0751, US Biomax, Inc.) for c-Myb. Positively stained cores are in red frame, negative cores are in black frame, low expression is identified in yellow frame.

Supplementary Table 1

Characteristics of tumor samples studied for myogenin and c-Myb expression by IHC

Tumor samples	Histology	Disease	Tumor status	IHC % myogenin +	IHC %c-Myb +	Outcome
1	ERMS	n.a.	n.a.	41	43	Alive
2	ARMS	Metastatic	P	70	80	Alive
3	ERMS	Localized	P	35	54	Alive
4	ERMS	Localized	P	35	46	Alive
5	ARMS	Metastatic	LR	58	58	Alive
6	ERMS	Localized	LR	10	20	Alive
7	ERMS	Localized	P	21	36	Alive
8	ERMS	Localized	LR	35	47	Alive
9	ARMS	Localized	P	45	49	Alive
10	ERMS	Metastatic	P	52	57	Alive
11	ARMS	Metastatic	LR	n.a.	n.a.	Alive
12	ARMS	Metastatic	P	67	66	DOD
13	Mixed ARMS/ERMS	Metastatic	P	54	62	Alive
14	ARMS	Metastatic	P	67	65	DOD
15	ARMS	Metastatic	P	63	62	Alive
16	ERMS	Localized	P	56	60	Alive
17	ERMS	Localized	P	10	9	Alive
18	ERMS	Metastatic	P	46	52	Alive
19	ERMS botryoid	Localized	P	4	4	Alive

Abbreviations: LR, local recurrence; P, primary; DOD, dead of disease

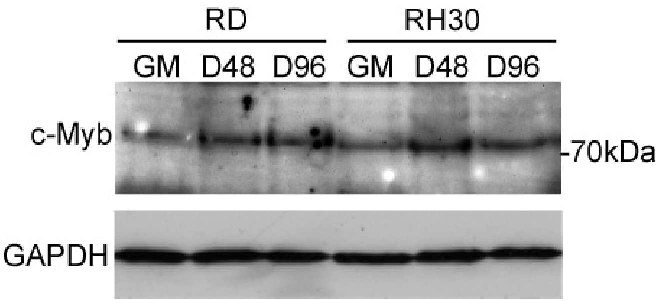

Supplementary Fig.3: RMS cell line RD and RH30 were cultivated in growth conditions (GM), differentiated for 48 hours (D48) and 96 hours (D96) and analyzed by western blotting for c-Myb expression.

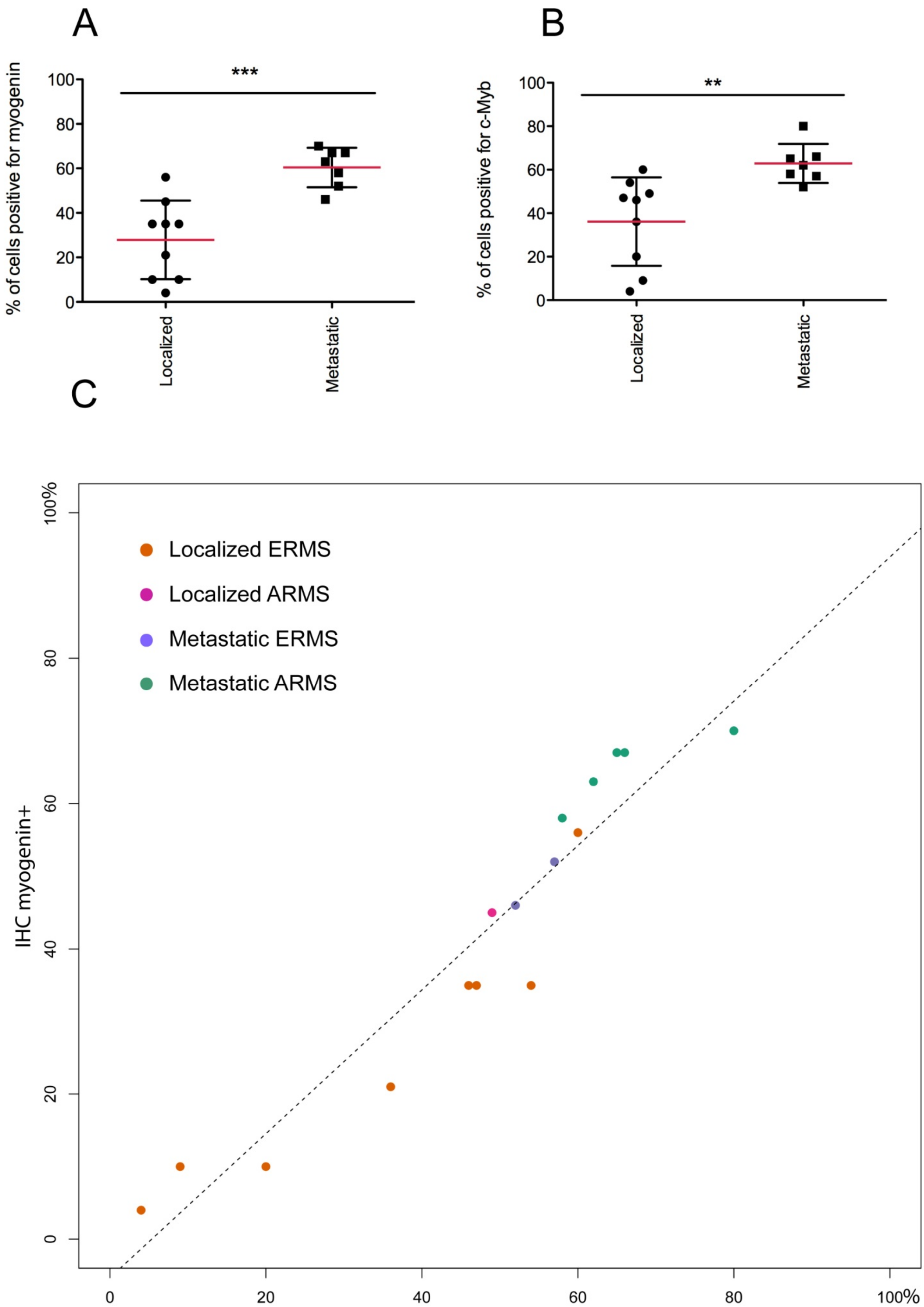

A
% of cells positive for myogenin
100
80
60
40
20
0
***
Localized
Metastatic
B
% of cells positive for c-Myb
100
80
60
40
20
0
**
Localized
Metastatic
C
Localized ERMS
Localized ARMS
Metastatic ERMS
Metastatic ARMS
IHC myogenin+
100%
80
60
40
20
0
IHC c-Myb+
0
20
40
60
80
100%

Supplementary Fig.4. Plots representing the percentage of myogenin positive cells (A) and percentage of c-Myb-positive cells (B) determined by immunohistochemistry in 9 localized RMS (8ERMS and 1ARMS) and 7 metastatic RMS (2ERMs and 5ARMS) (** P< 0.01,*** P< 0.001) as summarized in Supplementary Table 1. (C) Spearman´s rank correlation of c-Myb positive cells (%) with myogenin positive cells (%) determined by immunhistochemistry for RMS tumor samples as summarized in Supplementary Table 1.
